# Functional and structural changes throughout the auditory system following congenital and early-onset deafness: implications for hearing restoration

**DOI:** 10.3389/fnsys.2013.00092

**Published:** 2013-11-26

**Authors:** Blake E. Butler, Stephen G. Lomber

**Affiliations:** ^1^Cerebral Systems Laboratory, Department of Physiology and Pharmacology, Brain and Mind Institute, University of Western OntarioLondon, ON, Canada; ^2^Cerebral Systems Laboratory, Department of Physiology and Pharmacology and Department of Psychology, National Centre for Audiology, Brain and Mind Institute, University of Western OntarioLondon, ON, Canada

**Keywords:** hearing loss, brain development, auditory cortex, cochlear prostheses, hearing restoration

## Abstract

The absence of auditory input, particularly during development, causes widespread changes in the structure and function of the auditory system, extending from peripheral structures into auditory cortex. In humans, the consequences of these changes are far-reaching and often include detriments to language acquisition, and associated psychosocial issues. Much of what is currently known about the nature of deafness-related changes to auditory structures comes from studies of congenitally deaf or early-deafened animal models. Fortunately, the mammalian auditory system shows a high degree of preservation among species, allowing for generalization from these models to the human auditory system. This review begins with a comparison of common methods used to obtain deaf animal models, highlighting the specific advantages and anatomical consequences of each. Some consideration is also given to the effectiveness of methods used to measure hearing loss during and following deafening procedures. The structural and functional consequences of congenital and early-onset deafness have been examined across a variety of mammals. This review attempts to summarize these changes, which often involve alteration of hair cells and supporting cells in the cochleae, and anatomical and physiological changes that extend through subcortical structures and into cortex. The nature of these changes is discussed, and the impacts to neural processing are addressed. Finally, long-term changes in cortical structures are discussed, with a focus on the presence or absence of cross-modal plasticity. In addition to being of interest to our understanding of multisensory processing, these changes also have important implications for the use of assistive devices such as cochlear implants.

## Introduction

A profound childhood hearing loss can have widespread, devastating consequences that impact a child and their family for a lifetime. Perhaps most importantly, hearing loss can prevent a child from acquiring spoken language, which has a number of subsequent developmental and psychosocial consequences (see Möeller, 2007 for review). Fortunately, interventions which bypass damaged peripheral structures have been developed that allow for the restoration of auditory input. In fact, if implanted within a sensitive period for normal development, children with cochlear implants typically go on to display expressive and receptive language skills similar to those of normal hearing children by the time they are school-aged (e.g., Svirsky et al., 2004). However, successful intervention requires that the remaining auditory structures are of sufficient anatomical integrity, and functional state. For example, while cochlear implants have been successfully applied in cases of cochlear degeneration, they require intact spiral ganglion neurons be present in order to function.

Much of what we know about the changes to auditory structures that result from deafness, and how these changes have informed the design of cochlear prostheses, has come from studies in animal models. Fortunately, the subcortical auditory system is highly conserved among mammals (e.g., Glendenning and Masterton, 1998), such that a number of animal models exist that can inform our understanding of its structure and function. Moreover, a number of *deaf* animal models exist which closely resemble common morphologies of human disease (e.g., BALB/c mice, *deafness* mice, deaf-white cats). However, it is important to note that changes in the anatomy and function of peripheral and central auditory structures depend highly upon a number of factors, including the time of onset of hearing loss and the specific nature of the impairment. This review aims to address changes that occur in response to bilateral, congenital or early-onset deafness. Other forms of deafness (e.g., late-onset, unilateral, frequency-specific, etc.) are associated with a wide variety of highly specific changes that are beyond the scope of this paper. Here we address the most common methods for acquiring deaf animal models, including some discussion surrounding whether the methods currently used to evaluate hearing impairment are sufficient. We then describe anatomical and physiological changes that occur following deafness, including structures within the cochlea, subcortical nuclei, and within auditory cortex. Finally, we discuss cross-modal reorganization that appears to follow hearing impairment with some consideration of potential mechanisms.

## Deafening methods

The breadth and variability of phenotypes, mutations, mechanisms, and pathways associated with heritable deafness in humans is remarkable (Raviv et al., 2011). Thus, a high degree of variability in animal models is necessary to begin to understand the structural and functional changes associated with deafness. As a result, a number of methods have been used to produce animal models of profound deafness. While each has unique advantages and limitations, the development of any reliable technique requires that certain criteria be satisfied. Firstly, to minimize between-subject differences that might complicate the interpretation of post-deafening interventions, variability in the outcome of the procedure within a given species should be minimal. Ideally, this would include both variability in functional outcomes (i.e., threshold elevation), as well as variability in associated pathology. While there is some variability in the threshold elevation required for a deaf model, researchers typically seek models with ABR thresholds in excess of 80 dB nHL across the frequency range tested (see the section titled Measuring Deafness for more on outcome measurement). In order to avoid frequency-specific complications, any pathology associated with the deafening procedure should be uniform along the length of the cochlea. Finally, in order to minimize trauma associated with the procedure, steps should be taken to ensure the general health of the animal both during the procedure, and during post-procedural care. Here, we present a list of techniques that have been successful in generating animal models of hearing impairment, along with some commentary on their benefits and shortcomings.

### Genetic models of hearing loss

Across mammalian species, a number of genes required for normal cochlear function have been identified. For example, mutations in at least six mouse genes (PAX3, SOX10, MITF, SLUG, EDN3, and EDNRB) cause hereditary auditory-pigmentary disorders that mimic Waardenburg syndrome in humans (Tachibana et al., 2003). Transgenic and knock-out mouse strains that over- or under-express these genes have provided useful models of heritable conditions relating to inner and outer hair cell dysfunction that result in hearing loss (see Avraham, 2003 for review). These models allow for examination of the auditory system in great detail, and help improve our understanding of how very small-scale anatomical changes are related to hearing loss. Unfortunately, the genetic heterogeneity of hearing loss in humans involves hundreds of genes, with the possibility of multiple mutations contributing to disease etiology in any given patient (see Raviv et al., 2011, for review). Thus, while often related to hereditary hearing disorders in humans, gene-targeted models can be overly specific [e.g., a mouse model of the rare X-linked genetic mutation leading to progressive hearing loss associated with Norrie Disease (Berger et al., 1996)], such that data from these animals may not be generalizable to a large population of people with hearing impairments of varied origins.

Other models take advantage of the high incidence rates of congenital deafness that have been observed in a number of mammalian species. Examples include white minks, which provide yet another model of deafness associated with Waardenburg syndrome (e.g., Sugiura and Hilding, 1970), as well as collies (e.g., Lurie, 1948), Dalmatians (e.g., Lurie, 1948; Niparko and Finger, 1997), and deaf white cats (e.g., Bergsma and Brown, 1971), each of which models congenital deafness associated with the complete breakdown of cochlear structures often seen in the Scheibe deformity in humans. Across modalities, there is evidence that even a small amount of patterned sensory input at a very young age can initiate a cascade of developmental changes that can drastically alter the subsequent function of sensory systems (e.g., Hubel and Wiesel, 1970; Chang and Merzenich, 2003). Blocking the auditory canals of hearing animals at an early age provides a model of deafness associated with malformation of the external ear. However, this method produces an insufficient model of complete auditory deprivation, as many sounds (particularly those with low-frequency energy) are still transmitted to the cochlea via bone conduction (e.g., Popescu and Polley, 2010). Thus, congenitally deaf models are necessary to study the auditory system in the absence of input. Moreover, models like the congenitally deaf cat are useful for the study of late onset hearing (e.g., as provided by a cochlear implant), as the auditory nerve is well-preserved compared to other methods of deafening (Shepherd and Martin, 1995; Leake et al., 1999).

### Physical destruction

A second method of obtaining deaf animal models involves the physical destruction of cochlear structures. This is typically accomplished by creating an opening in the cochlea either by drilling through the cochlear wall (e.g., Sanes et al., 1992; Illing et al., 1997, 2005; Vale and Sanes, 2002), or by penetrating the round window (e.g., Tierney et al., 1997). Once exposed, the contents of the cochlea can be ablated using one of a number of small tools, and aspirated with a hollow glass needle. In the most extreme cases, the entire cochlea may be crushed with forceps and the remains aspirated (e.g., Rubio, 2006; Alvarado et al., 2009). One advantage of physical ablation is that the basilar membrane can be selectively lesioned, such that degeneration of spiral nerve fibers is largely restricted to the damaged area (Leake-Jones et al., 1982). This allows for the generation of models of partial hearing loss. However, the interpretation of pathology associated with cochlear implantation following physical ablation (Xu et al., 1993) and the growth of new bone (Leake-Jones et al., 1982) make this type of model impractical for studies of electrical stimulation of auditory nerve fibers.

An alternative method of physically ablating cochlear structures is through exposure to high-intensity sound, often in excess of 100 dB SPL (e.g., Sullivan et al., 2011). Such exposure can cause permanent damage to the cochlea that may provide a good model of frequency-specific hearing loss, such as the high-frequency impairments common in aging populations. However, the hearing loss produced is highly variable between individual animals (Bredberg, 1973; Cody and Robertson, 1983), such that the utility of noise-induced hearing loss for deafening animal models is limited.

It is also possible to prevent auditory stimuli from reaching subcortical and cortical auditory structures via bilateral transection of the auditory nerve. Unfortunately, efforts to completely transect the auditory nerve often cause inadvertent damage to adjacent vestibular nerve fibers. Conversely, overly conservative transections may preserve some auditory fibers, maintaining a partial representation of the cochlea.

### Ototoxic drug administration

The final class of methods used to obtain deaf animal models takes advantage of the ototoxic side-effects of common drugs. For example, their impressive efficacy and low cost make aminoglycoside antibiotics the most widely used class of antibacterial drugs worldwide (Forge and Schacht, 2000). However, their nephrotoxic and ototoxic side-effects have been well-documented. While some of the aminoglycosides (e.g., gentamycin, tobramycin, streptomycin) have been shown to be predominantly vestibulotoxic, others (e.g., neomycin, kanamycin, amikacin, dihydrostreptomycin) exhibit toxicity primarily within the cochlea. Across a number of species, the onset of this toxicity has been shown to be related to the onset of auditory function. For example, both rats (O'leary and Moore, 1992) and cats (Shepherd and Martin, 1995) receiving ototoxic drug administrations before the onset of hearing later showed normal auditory thresholds, while animals treated after the onset of hearing showed profound hearing losses. There also appears to be a sensitive period following the onset of hearing, during which animals are particularly sensitive to aminoglycoside toxicity (Henley and Rybak, 1995; Henley et al., 1996). During this period [post-natal days 11–16 in the rat (Henley et al., 1996)], decreased elimination rate constants, and increased half-lives lead to much higher mean serum aminoglycoside levels in young animals than in old animals. Thus, ototoxicity is expressed 2–3 times more quickly in these younger animals (Osaka et al., 1979; Astbury and Read, 1982).

The exact mechanisms involved in aminoglycoside ototoxicity are not fully understood. Labeled aminoglycosides appear first in the stria vascularis (Wang and Steyger, 2009), suggesting that they enter the fluids of the inner ear via strial capillaries, and subsequently accumulate in hair cells. The point of entry of aminoglycosides into cochlear hair cells is also not clear. While there is some suggestion that endocytosis is the primary mechanism (Hashino and Shero, 1995; Richardson et al., 1997), others advocate for the mechano-electrical transducer channel located on the stereocilia (Marcotti et al., 2005; Waguespack and Ricci, 2005). Still others suggest transient receptor potential channels expressed in the cochlea and permissive to aminoglycosides in renal cells may play a role (see Huth et al., 2011 for review). Within the hair cells, there is some speculation that mitochondria are the target of aminoglycoside toxicity. A maternally-linked genetic predisposition to ototoxic susceptibility (Hu et al., 1991) and the potentiation of toxicity that results from the inhibition of mitochondrial protein synthesis (Hyde, 1995), suggest that the drugs target and impair the function of mitochondrial RNA. This might explain why the toxic effects of aminoglycosides are readily observed in mitochondria-rich tissues like the organ of Corti.

Regardless of the mechanisms involved, it is clear that ototoxic aminoglycosides, when administered in sufficient quantity, can be used to produce deaf animals across a number of mammalian species. Repeated intramuscular injections of an aminoglycoside result in bilaterally symmetric hearing loss that progresses from high to low frequencies (Simmons et al., 1960). The time course of this hearing loss has been described as biphasic, consisting of a dramatic reduction in high frequency hearing occurring within 48 h of the first injection, followed by a slow reduction that proceeds from high to low frequencies over several weeks (Shepherd and Clark, 1985). While effective, there is considerable variability in the extent of cochlear damage between individuals for any given drug dosage (Leake-Jones et al., 1982). Repeated drug administrations of this nature are also stressful to the animal, and time consuming for the experimenter (e.g., the cats deafened by Shepherd and Clark (1985) first showed profound low-frequency hearing loss 75 days following drug administration). Finally, the risk of kidney failure following repeated drug administrations is significant and thus, renal function must be constantly monitored during the deafening procedure.

In an effort to circumvent systemic effects, alternative methods involve aspirating the cochlear lymph, and administering aminoglycoside antibiotics directly to the cochlea (e.g., Leake-Jones et al., 1982; Zettel et al., 2003; Asako et al., 2005). This method causes the rapid-onset of profound hearing loss across the entire frequency range. While this method may prevent undesirable nephrotoxicity, the degree of destruction in the organ of Corti is extreme and may limit the types of deafness that can be modeled in this manner. Moreover, this method is not ideal for studies of cochlear implant function, as it can result in extensive fibrous tissue and bone growth within the scala tympani (Sutton and Miller, 1983).

A promising method for deafening animals involves administering an aminoglycoside antibiotic in combination with an infusion of a loop diuretic, such as furosemide or ethacrynic acid (West et al., 1973). Loop diuretics do not result in permanent ototoxicity when administered in isolation; rather, they are thought to act on the stria vascularis to reduce the endocochlear potential, causing a subsequent alteration of the ionic composition of the endolymph that fills the scala media. Typically, a single injection of an aminoglycoside is given enough time to accumulate in the cochlea. A loop diuretic is then infused, and the animal's hearing thresholds are either continuously monitored, or periodically monitored, usually via auditory brainstem responses (ABR). This method produces a rapid and dramatic bilateral hearing loss in guinea pigs (West et al., 1973; Brummett et al., 1979) and cats (Xu et al., 1993). Unfortunately, efficacy differs by species, with the same procedure resulting in only mild hearing loss, and acute renal failure in the macaque (Shepherd et al., 1994).

## Measuring deafness

Regardless of the procedure used, successful deafening depends on valid and reliable methods of measuring the degree of hearing loss achieved. For example, one early method of assessing deafness that is now rarely used involved producing a loud hand clap and observing whether an animal responded with reflexive movement of the pinnae or a startle reflex (Preyer, 1882). While the absence of Preyer's reflex has been shown to correlate well with profound hearing loss (Jero et al., 2001), the method relied heavily on subjective measure, and was incapable of distinguishing between conductive and sensorineural hearing loss.

**Figure 1:**
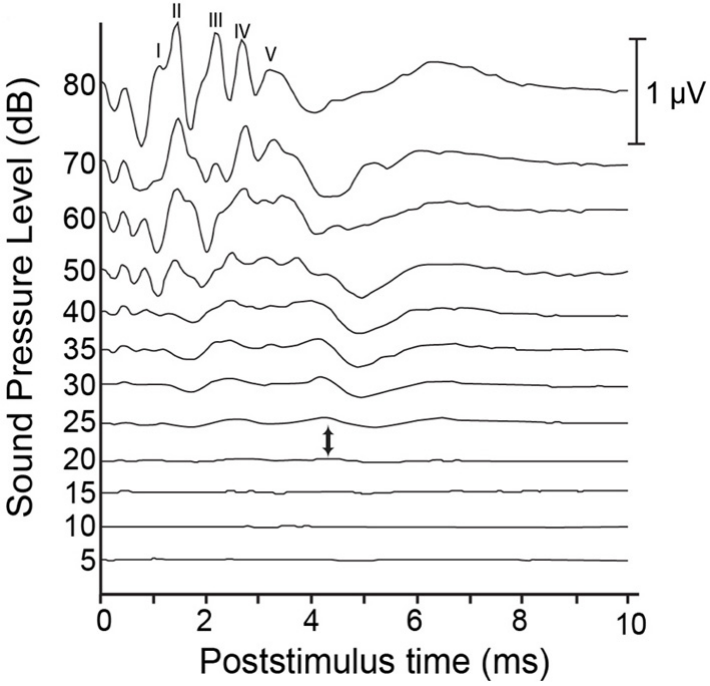
**Sample auditory brainstem responses (ABR) from a hearing cat.** Auditory clicks are presented at levels ranging from 80 dB down to 5 dB SPL. Responses represent the average of 1000 presentations, and are comprised of 5 peaks: wave I is thought to be generated by the peripheral auditory nerve; wave II by the central auditory nerve; wave III by the cochlear nucleus; wave IV by the superior olive and lateral lemniscus; and wave V by the lateral lemniscus and inferior colliculus. Each of these characteristic peaks shows a reduction in amplitude and an increase in latency as presentation level decreases. Auditory thresholds are typically considered to lie somewhere between the presentation level at which no discernible response is present and the level at which a response is first elicited (between 20 and 25 dB, respectively, in this example).

Currently, an overwhelming majority of researchers rely on an auditory brainstem response (ABR) to define the endpoint of, or to measure the success of their deafening procedure. ABRs can be evoked using a variety of auditory stimuli. However, researchers typically rely on click-evoked ABRs to measure deafness in animal models (see Figure 1 for an example). While clicks contain energy across a wide frequency band, click-evoked ABRs are not equally sensitive to hearing loss across this same range. Rather, the results of click-evoked ABRs in humans correlate best on average with audiometric thresholds in the range of 2–4 kHz (Watkins and Baldwin, 1999). Indeed, Shepherd and Martin (1995) noted that the click-evoked ABR is not a good predictor of high-frequency hearing loss in cats; such losses are better revealed by ABR audiograms that measure thresholds at different pure tone frequencies. Insensitivity of click-evoked ABRs to high frequency hearing loss is cause for concern when monitoring the auditory status of humans receiving aminoglycoside antibiotics, as this class of drugs is known to first impair hair cell function in the high frequency range (Simmons et al., 1960), and thus, early signs of hearing loss may be masked. However, when deafening an animal via ototoxic drug administration, the goal of the procedure is often profound hearing loss across all frequencies. Thus, the correlation between low-frequency hearing and click-evoked ABR results is less troublesome. That being said, pure tone-evoked ABR provides an alternative method for measuring hearing thresholds across a variety of frequencies, provided that the frequencies chosen represent the extent of the audible frequency range of the animal in question.

In humans, the click-evoked ABR is a widely used screening tool for hearing loss in neonates (see Hyde, 2005 for review). Typically, an automated screening device provides a pass/fail output with no need for subjective evaluation, but also produces false-positive rates between 3 and 8% (Barsky-Firkser and Sun, 1997; Mason and Herrman, 1998; Mehl and Thompson, 1998). Infants who fail this initial screen are referred for further audiological assessment. In addition to deafness, a number of disease conditions can result in abnormal ABRs, including posterior fossa tumors, vertebra-vascular pathology, demyelinating diseases, central nervous system infections, and polyneuropathies (Thomsen and Tos, 1990). In humans, diagnosis of the cause of ABR abnormality requires follow-up audiometric testing and imaging. Fortunately, follow-up measures can be avoided when using ABR to assess the success of deafening, provided the animal was shown to have a normal ABR at the onset of the procedure. When deafening animal models, hearing loss is typically considered to be complete when waves I through V of the ABR are absent at stimulus intensities of 80 dB nHL or greater. While this subjective evaluation may be a cause for concern, the complete absence of wave I reflects a lack of activity in the auditory nerve (e.g., Starr, 1976) and thus, should be expected to reflect profound hearing loss throughout the central and peripheral auditory structures.

In sum, the ABR represents a quick and inexpensive method of monitoring auditory system function during or following deafening procedures. Click-evoked ABRs may be insensitive to high-frequency hearing losses that often precede impairment at lower frequencies in aminoglycoside-induced deafness. However, when seeking to ensure a profound hearing loss across all frequencies, ABR is well-suited, provided that a baseline ABR is suggestive of normal hearing status prior to deafening.

## Effects of deafness on the auditory system

### The cochlea and cochlear nerve

**Figure 2:**
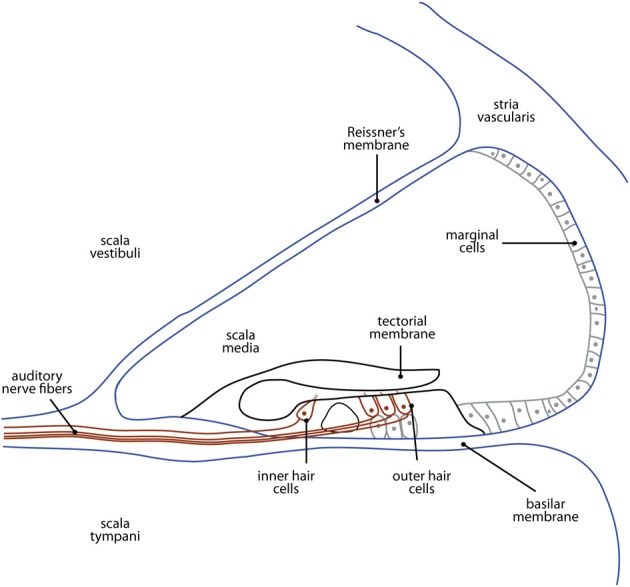
**A cross-sectional illustration of the cochlea showing the structures most commonly affected in animal models of deafness.** The etiology of hearing loss depends on the type of model used, and is described in detail in the section titled The Cochlea and Cochlear Nerve.

The nature of the cochlear damage involved with hearing loss in animal models differs widely depending on the nature of the deficit. For example, in the case of mechanical destruction of the cochlea, the impact to cochlear structures is decidedly dependent on the extent of damage (see Figure 2 for an illustration of cochlear structures commonly affected). Conversely, the cochlear damage associated with genetic models of hearing loss is particular to the specific genes involved. For example, deaf white cats mimic the Scheibe deformity in humans, presenting with early-onset, progressive cochleosaccular degeneration and severe sensorineural hearing impairment (Scheibe, 1892). However, the rate and extent of pathology are widely variable between animals. The traditionally described course of pathology involves cochleosaccular degeneration that begins at the end of the first postnatal week with the sagging and ultimate collapse of Reissner's membrane, distortion of the tectorial membrane, and atrophy of the stria vascularis (Bosher and Hallpike, 1965, 1967; Ryugo et al., 1997, 1998, 2003). However, additional forms of pathology have been described involving excessive epithelial growth within the bony labyrinth either in isolation, or in addition to the collapse of Reissner's membrane (Ryugo et al., 2003; Baker et al., 2010). In any case, these anatomical changes are typically followed by hair cell destruction that proceeds from the cochlear base toward its apex (e.g., Leake et al., 1997; Ryugo et al., 1998), mimicking the pattern of maturation in the organ of Corti (Pujol and Marty, 1970; Romand and Romand, 1982; Lim and Anniko, 1984). The extent of cell loss ranges from the basal 20% of the cochlea in cases of threshold elevation (single unit thresholds in excess of 60 dB SPL for tones below 10 kHz; Ryugo et al., 1998) to the eventual complete loss of inner and outer hair cells, as well as supporting cells along the entire length of the basilar membrane in cases of complete deafness (Rebillard et al., 1981; Ryugo et al., 1998).

Each inner hair cell of the cochlea has a direct, one-to-one connection with a spherical bushy cell in the cochlear nucleus, via a type I spiral ganglion neuron (SGN; Sento and Ryugo, 1989). The number of SGNs is drastically reduced in hearing impaired animals; the nature of the deficit appears to depend on the degree and duration of hearing loss, as well as the age at which it occurs. In animals with congenitally elevated thresholds (Ryugo et al., 1998), damage is often limited to those cells which innervate the most basal portion of the cochlea. Short-term deafened adult animals also show maximal cell loss in basal SGNs (Leake and Hradek, 1988), while animals deafened during development, before the onset of hearing, present maximal SGN degeneration in a region approximately 40-60% from the cochlear base (Leake et al., 1991, 1992). Finally, congenitally deaf (Ryugo et al., 1998) and long-term deafened animals (Shepherd et al., 2004; Hurley et al., 2007) present with a dramatic reduction in SGNs throughout the entirety of the cochlea. The process of SGN loss begins with the loss of unmyelinated peripheral processes in the organ of Corti, followed by a gradual degeneration of myelinated processes in the spiral lamina, and of the cell somata within Rosenthal's canal (Leake and Hradek, 1988; Heid et al., 1998; Hardie and Shepherd, 1999). Surviving SGNs are devoid of a perikaryal myelin sheath (Leake and Hradek, 1988; Shepherd and Hardie, 2001), which can lead to reduced temporal resolution (Zhou et al., 1995), increased refractory properties (Shepherd et al., 2004), and evidence of conduction block (Shepherd and Javel, 1997). Schwann cells within the deafened cochlea can survive for some time despite the degeneration of SGNs, however, there is some evidence that they revert to a non-myelinating phenotype (Hurley et al., 2007).

It has been suggested that alterations of SGN structure occur secondary to cochlear pathology in a variety of species, including deaf white cats (Bosher and Hallpike, 1965, 1967; Suga and Hattler, 1970; Mair, 1973; West and Harrison, 1973; Elverland et al., 1975), mice (Mikaelian et al., 1974), Dalmatians (Johnsson et al., 1973; Mair, 1976), and humans (Altmann, 1950). There is some evidence that the survival of SGNs depends on endogenous, pro-survival neurotrophin peptides that are normally provided by the hair cells and supporting cells of the cochlea (Springer and Kitzman, 1998; Landry et al., 2011). However, others have suggested that SGN pathology represents a separate degenerative process that can precede or follow cochlear damage (Pujol et al., 1977; Leake et al., 1997). Indeed, in some cases the pattern of SGN loss differs significantly from the pattern of cochlear pathology, lending support to this latter view. Furthermore, in congenitally deaf animals, a large number of unmyelinated SGNs are found before evidence of other sensory or epithelial deficits occur, and in some cases SGN degeneration precedes damage to the sensory cells of the cochlea (Pujol et al., 1977).

Auditory nerve fibers bifurcate in the ventral cochlear nucleus, sending an ascending branch rostrally in the anterior division (AVCN), and a descending branch caudally into the posterior division (PVCN) of the ventral nucleus, which ultimately innervates the dorsal nucleus. These branches terminate in a variety of structures including endbulbs of Held, modified endbulbs, and terminal boutons which may be accompanied by a series of en passant swellings. In normal hearing animals, endbulbs of Held typically exhibit a complex arborization, with multiple branches that stem from a single, thick trunk. These endings typically contact up to half of the soma of a spherical bushy cell (SBC; Ryugo et al., 1997). In contrast, the endbulbs of Held that remain following deafness exhibit less extensive arborization, giving rise to fewer en passant and terminal swellings that are larger in size, and which contain fewer synaptic vesicles on average than those of normal hearing animals (Ryugo et al., 1997, 1998; Limb and Ryugo, 2000; Lee et al., 2003; Baker et al., 2010; O'Neil et al., 2011). The fine, interconnected varicosities and branches present in the endbulbs of Held of normal hearing animals are absent in the deaf, leading to diminished contact with the target bushy cell (Ryugo et al., 1998). In fact, evidence of morphological differences between the endbulbs of Held of deaf animals and those of hearing animals are evident at birth both in deaf white cats (Baker et al., 2010) and mice (Oleskevich and Walmsley, 2002; McKay and Oleskevich, 2007). In contrast, the modified endbulbs of deaf animals, which typically contact globular bushy cells (GBCs) in the VCN, show a drastic reduction in size, but are not different from those of normal hearing animals in terms of complexity (Redd et al., 2000). Finally, the bouton endings that synapse on multipolar cells of the cochlear nucleus are significantly smaller in congenitally deaf animals than in normal hearing controls (Redd et al., 2002).

The highly-organized pattern of neurons projecting to the cochlear nucleus helps maintain the tonotopic organization initiated in the cochlea. In hearing animals, these projections are broad prior to the onset of hearing, and are refined during a sensitive period for development occurring shortly thereafter (Snyder and Leake, 1997). However, this refinement is activity-dependent; the tonotopic specificity of projections to the cochlear nucleus is significantly degraded in hearing impaired animals (Leake et al., 2006).

### Subcortical nuclei

#### Cochlear nucleus (Table 1)

**Figure 3:**
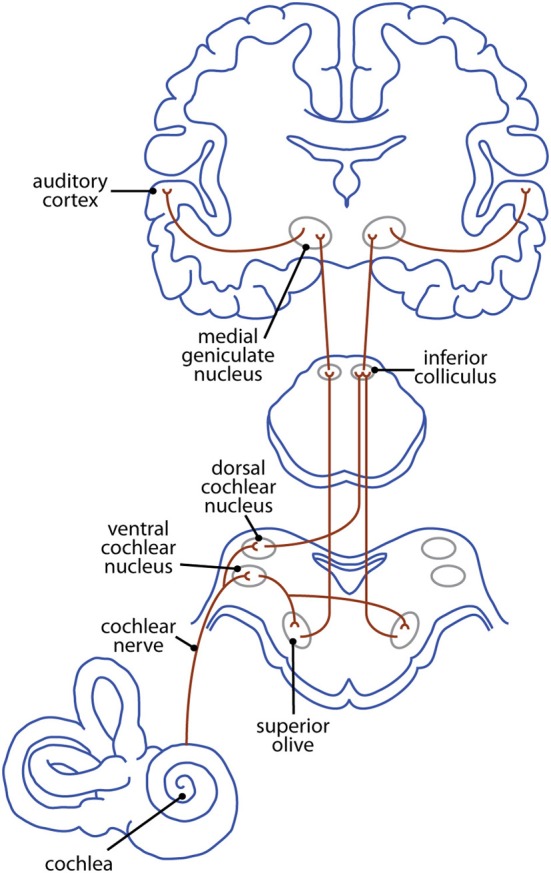
**The ascending mammalian auditory pathway—from cochlea to cortex**.

In many ways, the *pattern* of ascending auditory projections in the brainstem of congenitally deaf animals appears normal (Heid et al., 1997). However, anatomical and functional changes are present at most levels of this pathway (Figure 3). The precise nature of these changes depends on a number of factors, including an animal's age at the onset of deafness and the intervention used to induce deafness. Thus, a summary table is provided for each of the following subcortical and cortical sections to allow for direct comparison across age and methodology.

**Table 1:** **Summary of changes in cochlear nuclei**.

**Author(s)**	**Species**	**Etiology**	**Deafness onset**	**Change(s) observed**
Nordeen et al., 1983	Gerbil	Cochlear ablation	Day 1–2	↓ # of neurons
Moore et al., 1998	Rats	Ototoxicity	Day 6–10	No change in # of neurons
		Cochlear removal	Day 6 Day 12	↓ # of neurons
				No change in # of neurons
Stakhovskaya et al., 2008	Cat	Ototoxic	Day 16–24	↓↓↓ volume
			Day 48–56	↓ volume
Moore and Kowalchuk, 1988	Ferret	Cochlear lesion	Day 12–93	↓ volume in dorsal division
				↓↓↓ volume in ventral divisions
				↓ size of bushy cell somata
Anniko et al., 1989	Mouse	Congenital	Day 0	↓ volume in dorsal division
				↓↓↓ volume in ventral divisions
Tierney et al., 1997	Gerbils	Cochlear removal	Day 3–7	↓ # of neurons
				↓↓↓ volume
				↓ neuron size
			Day 11–93	No change in # of neurons
				↓ volume
				↓ neuron size
Hashisaki and Rubel, 1989	Gerbils	Cochlear removal	Day 7	↓ # of neurons
				↓ size of neurons
			Day 140	No change in # of neurons
				↓ size of neurons
Hulcrantz et al., 1991	Cat	Ototoxic	Day 14–16	↓ size of neurons
Lustig et al., 1994	Cat	Ototoxic	Day 14–16	↓ size of neurons
Saada et al., 1996	Cat	Congenital	Day 0	↓ size of neurons
Hardie and Shepherd, 1999	Cat	Ototoxic	Day 10	↓ size of neurons
Saada et al., 1996	Cat	Congenital	Day 0	↓ size of bushy cell somata
West and Harrison, 1973	Cat	Congenital	Day 0	↓ size of bushy cell somata
Redd et al., 2002	Cat	Congenital	Day 0	↓ size of multipolar cell bodies
				↓ complexity of synaptic cleft
Redd et al., 2000	Cat	Congenital	Day 0	PSDs are flattened and ↓ in size
				↑ in neurotransmitter receptors
Ryugo et al., 2010	Cat	Ototoxic	Day 17–24	PSDs are flattened and ↑ in size
Oleskevich and Walmsley, 2002	Mouse	Congenital	Day 0	↑ neurotransmitter release probability
Wang and Manis, 2006	Mouse	Congenital	Day 20–57	↓ temporal resolution

PSD, Postsynaptic density, Day 0 = Day of birth.

Neonatal removal of the cochlea or blockade of cochlear nerve activity results in reduced cochlear nucleus volume resulting from decreases in the number of neurons (Nordeen et al., 1983; Tierney et al., 1997; Moore et al., 1998), in the size of neurons (Hulcrantz et al., 1991; Lustig et al., 1994; Saada et al., 1996; Hardie and Shepherd, 1999), or a combination of the two (Hashisaki and Rubel, 1989). The magnitude of these changes is dependent on a number of factors including the degree of ganglion cell loss (Moore and Kowalchuk, 1988; Hardie and Shepherd, 1999), and the duration of hearing impairment (Hardie and Shepherd, 1999). Additionally, changes in both neuronal size and number appear to be related to the time at which auditory input is removed; the greatest decrease occurs when animals are deafened long before the onset of hearing, while those animals deafened at or after hearing onset show no change (Tierney et al., 1997; Stakhovskaya et al., 2008). Finally, the decrease in volume is typically less severe in the dorsal division than in either of the ventral divisions (Moore and Kowalchuk, 1988; Anniko et al., 1989; but see Saada et al., 1996).

In the cochlear nuclei of hearing animals, post-synaptic densities (PSDs) cover the somata of bushy cells. These PSDs are punctate and tend to present as distinct convexities that indent the presynaptic membrane (Redd et al., 2000). In contrast, bushy cells of congenitally deaf animals contain PSDs that are larger and appear flattened (Redd et al., 2000; Ryugo et al., 2010), while the cell somata themselves are smaller than those of hearing animals (West and Harrison, 1973; Moore and Kowalchuk, 1988; Saada et al., 1996). While no deafness-related changes in the size of PSDs, or in the synaptic vessel density have been reported for multipolar cells in the cochlear nucleus, the cell bodies themselves are significantly smaller in deaf animals than in normal hearing controls (Redd et al., 2002). Additionally, the system of channels that exists in the synaptic cleft between these cells and the terminal boutons of ascending auditory nerve fibers (which likely functions to remove neurotransmitter from the synapse) is significantly less complex following hearing loss (Redd et al., 2002).

In addition to these anatomical differences, changes in the function of synapses in the cochlear nucleus appear to increase the likelihood of action potential generation under conditions of drastically diminished spike activity. For example, some deaf models show an increase in neurotransmitter release probability, relative to normal hearing controls (Oleskevich and Walmsley, 2002). Concurrently, hypertrophy of PSDs in the deaf cochlear nucleus may represent an upregulation of the neurotransmitter receptors in order to optimize potential responses (Redd et al., 2000). It has been suggested that the differential effects of deafness on PSD size and vesicle density between bushy cells and multipolar cells may be related to the baseline activity levels; those cells which are normally highly active (bushy cells) undergo large-scale compensatory changes following deafness, while cells with lower baseline rates of activity (multipolar cells) undergo little or no change (Redd et al., 2002). It has further been suggested that the changes occurring at bushy cell synapses may impair the ability of those cells to reliably preserve temporal coding information arriving from ganglion cells (Wang and Manis, 2006).

#### Superior olive (Table 2)

The superior olivary complex consists of three primary nuclei, the medial superior olive (MSO), lateral superior olive (LSO), and the medial nucleus of the geniculate body (MNTB), along with several smaller periolivary nuclei. In hearing animals, the MSO, LSO, and MNTB contribute to sound localization in the azimuth, and are tonotopically organized. Rough frequency gradients in these nuclei are established by the differential expression of ion channels (e.g., Li et al., 2001) and currents (e.g., Leao et al., 2006) along the tonotopic axis, occurring before the onset of hearing. However, these physiological gradients are dependent on spontaneous activity in the cochlear nerve, and fail to develop in congenitally deaf models that lack spontaneous spiking (von Hehn et al., 2004; Leao et al., 2006).

**Table 2:** **Summary of changes in superior olivary nuclei**.

**Author(s)**	**Species**	**Etiology**	**Deafness onset**	**Change(s) observed**
Russell and Moore, 1999	Gerbil	Cochlear removal	Day 18	↓ # of dendrites in MSO
Schwartz and Higa, 1982	Cat	Congenital	Day 0	↓ MSO neuron size
				↓ size of the MSO
Tirko and Ryugo, 2012	Cat	Congenital	Day 0	No change in MSO neuron size
				No change in nucleus size
				↓ terminal bouton size in MSO
				↓ inhibitory input at MSO cell soma/dendrites
Kapfer et al., 2002	Gerbil	Noise exposure	Day 10–25	↓ inhibitory input at MSO cell soma
Moore, 1992	Ferret	Cochlear removal	Day 5	↓ # of LSO neurons
				↓ size of the LSO
Pasic et al., 1994	Gerbil	Cochlear ablation	Day 28–42	↓ size of MNTB neurons
Oleskevich and Walmsley, 2002	Mice	Congenital	Day 0	Calyx of Held matures normally
Oleskevich et al., 2004	Mice	Congenital	Day 0	Calyx of Held matures normally
Youssoufian et al., 2005	Mice	Congenital	Day 0	Calyx of Held matures normally
Leao et al., 2006	Mice	Congenital	Day 0	Tonotopy is disrupted
von Hehn et al., 2004	Mice	Congenital	Day 0	Tonotopy is disrupted

MSO, Medial superior olive; LSO, Lateral superior olive; MNTB, Medial nucleus of the trapezoid body.

The MSO receives input from the cochlear nuclei bilaterally. Within the MSO of hearing animals, excitatory inputs are segregated such that ipsilateral inputs terminate on lateral dendrites while contralateral inputs terminate medially (Russell and Moore, 1995; Kapfer et al., 2002). In the absence of auditory input, dendrites of MSO neurons have been shown to undergo selective atrophy, leading to a reduction in the number, but not in the overall area of dendritic profiles (Russell and Moore, 1999). Some researchers report age-related decreases in the size of MSO neurons, and the total volume of the nucleus of congenitally deaf animals (Schwartz and Higa, 1982), while others fail to find evidence for such changes (Tirko and Ryugo, 2012). Inhibitory inputs of normal hearing mammals specialized for low frequency hearing (e.g., gerbil, cat, chinchilla) tend to be confined primarily to MSO cell bodies (Werthat et al., 2008; Couchman et al., 2010). This spatial arrangement is thought to be crucial for processing the sub-millisecond interaural differences that allow for accurate sound localization, and is the result of neural activity-dependent developmental change. Deafness causes a bilateral disruption in the spatial segregation of MSO neurons, with a significant reduction in inhibitory input at the cell somata (Kapfer et al., 2002; Tirko and Ryugo, 2012) and along the dendrites (Tirko and Ryugo, 2012). While the density of terminations on MSO cell dendrites does not change following hearing loss, the terminal boutons of deaf animals are significantly smaller than those of normal hearing animals (Tirko and Ryugo, 2012).

The LSO receives excitatory input from the ipsilateral cochlear nucleus, and inhibitory input from the contralateral cochlear nucleus, via the MNTB. In animal models of hearing loss, cochlear destruction leads to neural loss and shrinkage of the LSO (Moore, 1992) and a decrease in the size of cell somata in MNTB (Pasic et al., 1994). Within the MNTB, there is a large central synapse known as the calyx of Held that undergoes remarkable development to ensure the high-fidelity transfer of sound information. Interestingly, this development appears to be unrelated to both spontaneous and sound-evoked neural activity, such that the calyx of Held matures normally in deaf animals (Oleskevich and Walmsley, 2002; Oleskevich et al., 2004; Youssoufian et al., 2005). In hearing animals, the rough tonotopy established before the onset of hearing is later refined such that each neuron of the LSO receives excitatory and inhibitory inputs from neurons that respond to the same sound frequency (Kandler et al., 2009). However, the pruning that leads to this sophisticated tonotopy depends in large part on auditory-evoked activity (Gillespie et al., 2005; Kandler et al., 2009) and fails to occur following early-onset deafness.

#### Inferior colliculus (Table 3)

The inferior colliculus (IC) is comprised of dorsal and lateral cortices that collectively form a “rind” surrounding the central core (Winer, 2005). This central nucleus receives inputs from the cochlear nuclei, superior olives, and nuclei of the lateral lemniscus, as well as descending inputs from the auditory cortex and superior colliculus. Interestingly, the pattern of projections remains relatively unchanged following long-term auditory deprivation. For example, projections from the cochlear nucleus to the ipsilateral IC show no change in number following bilateral cochlear removal (Moore, 1990), while projections to the contralateral IC show either a small decrease (Trune, 1983) or no change at all (Moore and Kowalchuk, 1988; Moore, 1994). Similarly, the number of projections from the cochlear nucleus to IC is unaffected by congenital deafness (Heid et al., 1997). Projections from the superior olivary complex to IC are similarly unaffected by cochlear removal (Russell and Moore, 1995) or congenital deafness (Heid et al., 1997). Finally, a rudimentary representation of tonotopy persists in the IC following long-term deafness (Snyder et al., 1990, 1991; Heid et al., 1997; Shepherd and Javel, 1999), suggesting that frequency-based organization is established independent of patterned auditory activity.

**Table 3:** **Summary of changes in inferior colliculi**.

**Author(s)**	**Species**	**Etiology**	**Deafness onset**	**Change(s) observed**
Moore and Kowalchuk, 1988	Ferret	Cochlear lesion	Day 12–93	↓ volume
Nishiyama et al., 2000	Cat	Ototoxic	Day 10	↓ soma area
Hardie et al., 1998	Cat	Ototoxic	Day 10	↓ synaptic density
				↓ # of presynaptic vesicles
Trune, 1983	Mouse	Cochlear lesion	Day 6	↓ # of projections from CN
Vale and Sanes, 2000	Gerbil	Cochlear ablation	Day 7	↓ inhibitory synapse strength
Vale and Sanes, 2002	Gerbil	Cochlear ablation	Day 9	↓ inhibitory and excitatory synapse strength
Shepherd et al., 1999	Cat	Ototoxic	Day 10	↓ temporal resolution
Snyder et al., 1995	Cat	Ototoxic	Day 16	↓ temporal resolution
Vollmer et al., 2005	Cat	Ototoxic	Day 14–25	↓ temporal resolution
Moore, 1994	Ferret	Cochlear removal	Day 25	No change in # of neurons
Moore, 1990	Ferret	Cochlear removal	Day 5	No change in # of neurons
				No change in projection pattern from CN
Russell and Moore, 1995	Gerbil	Cochlear removal	Day 2–14	No change in projection pattern from SO
Heid et al., 1997	Cat	Congenital	Day 0	No change in projection pattern from CN/SO
				Tonotopy is maintained
Shepherd and Javel, 1999	Cat	Ototoxic	Unknown	Tonotopy is maintained
Snyder et al., 1990	Cat	Ototoxic	Day 16	Tonotopy is maintained
Snyder et al., 1991	Cat	Ototoxic	Day 16	Tonotopy is maintained

CN, Cochlear nucleus; SO, Superior olive.

While the number of projections to the IC appears unaffected by hearing loss, there appear to be substantial qualitative differences between IC neurons in hearing-deprived and normal hearing animals. The somata of IC neurons in bilaterally deafened animals undergo some degree of atrophy, resulting in a slight but significant decrease in soma area relative to normal hearing controls (Nishiyama et al., 2000). Moreover, early-onset hearing loss leads to a sharp reduction in synaptic density relative to normal hearing animals, and an apparent decrease in the number of presynaptic vesicles in many of the remaining synapses (Hardie et al., 1998). Developmental studies have shown that dramatic increases in synaptic density in the IC follow the onset of hearing, suggesting a role for stimulus-evoked neural activity in shaping connections in this nucleus (Aitkin et al., 1996, 1997).

Functionally, bilateral cochlear ablation causes a rapid loss of inhibitory (Vale and Sanes, 2000, 2002) and excitatory (Vale and Sanes, 2002) synaptic strength, as a result of changes to both pre- and post-synaptic mechanisms. Vale and Sanes (2002) have demonstrated that these changes are independent of deafferentation-induced cell death of neurons in the cochlear nucleus. IC neurons deprived of auditory input also demonstrate poor temporal resolution, with decreased maximum following frequencies and longer response latencies than IC neurons in normal hearing animals (Snyder et al., 1995; Shepherd et al., 1999; Vollmer et al., 2005).

#### Medial geniculate body

The medial geniculate body (MGB) is the auditory thalamic processing station between the inferior colliculus and the auditory cortex. Across species, the MGB is typically subdivided into multiple subsections, each of which contains several nuclei that process both afferent and efferent neural activity (Winer, 1984; Clerici and Coleman, 1990). Despite its importance to the auditory system, there is a paucity of information on changes at the thalamic level following deafness; a single study has identified normal cortical projections to A1 from the MGB of neonatally deafened animals (Stanton and Harrison, 2000). There are a number of potential reasons for this lack of information, the most likely of which is difficulty accessing thalamic structures. Because of its location, the MGB is very difficult to target, both for neuroanatomical tracer injection, and for the type of *in vivo* electrophysiological studies that have measured function at other levels of the auditory system. Changes in the pattern of projection to auditory cortex could be revealed through cortical injections of retrograde tracers; however, these studies have not yet been undertaken.

### Auditory cortex (Table 4)

The primary auditory cortex (A1) is the most extensively studied area of auditory cortex. In congenitally deaf animals, A1 has a similar laminar structure to that of hearing animals (Hartmann et al., 1997). Electrophysiological studies have suggested that the area occupied by A1 increases slightly following neonatal deafening (Raggio and Schreiner, 1999), while the size of A1 in congenitally deaf animals appears to be no different than in hearing animals (Kral et al., 2002). However, anatomical studies demonstrate that auditory cortex decreases in size following hearing loss, and that this decrease is correlated with the age of deafness onset (Wong et al., 2013a). In particular, the size of A1 appears drastically reduced following early-onset deafness (Wong et al., 2013a) as well as in congenitally deaf animals (Wong et al., 2013b). In addition, congenitally deaf animals present with reductions in both the number of primary dendrites and in the span of dendritic trees in primary auditory cortex relative to hearing controls (Kral et al., 2006). Thus, while gross level anatomical similarities may exist between hearing and non-hearing animals, functional connectivity differs greatly between the two. For example, inputs to layers III/IV of A1 are present in congenitally deaf animals, as are subsequent inputs to more superficial, supergranular layers (Klinke et al., 1999). However, activity in deeper, infragranular layers is significantly decreased (Kral et al., 2000, 2002), and synaptic current latencies are significantly longer [after controlling for brainstem latency shifts (Kral et al., 2000; Klinke et al., 2001)], suggesting that connections between superficial and deeper layers do not mature. In hearing animals, the infragranular layers of A1 are the source of descending, feedback projections. Thus, inactivity in these layers following auditory deprivation suggests that subcortical feedback loops are likely non-functional.

**Table 4:** **Summary of changes in auditory cortices**.

**Author(s)**	**Species**	**Etiology**	**Deafness onset**	**Change(s) observed**
Raggio and Schreiner, 1999	Cat	Ototoxic	Day 16–21	↑ in area of A1
				↑ excitability/↓ inhibition of A1 neurons
				No change in rate- or latency-intensity functions
				Tonotopy is lost
Kral et al., 2002	Cat	Congenital	Day 0	No change in size of A1
				Immature connections to deeper layers
				Coarse tonotopy maintained
Wong et al., 2013a	Cat	Ototoxic	Early late	↓↓↓ in area of A1
				↓ in area of A1
Wong et al., 2013b	Cat	Congenital	Day 0	↓↓↓ in area of A1
Kral et al., 2006	Cat	Congenital	Day 0	↓ in # of primary dendrites
				↓ in span of dendritic trees
Hartmann et al., 1997	Cat	Congenital	Day 0	No change in laminar structure
Stanton and Harrison, 2000	Cat	Ototoxic	Day 6	No change in thalamocortical projections to A1
Klinke et al., 1999	Cat	Congenital	Day 0	Inputs to layers III/IV remain
				Connections to supergranular layers remain
Kral et al., 2000	Cat	Congenital	Day 0	Immature connections to deeper layers
Kral et al., 2003	Cat	Congenital	Day 0	↑ spontaneous firing rate
Kotak et al., 2005	Gerbil	Cochlear ablation	Dat 10	↑ excitability/↓ inhibition of A1 neurons
Kral et al., 2005	Cat	Congenital	Day 0	↑ excitability/↓ inhibition of A1 neurons
				↓ response to electrical stimulation
Raggio and Schreiner, 1994	Cat	Ototoxic	Day 16–21	No change in rate- or latency-intensity functions
Tillein et al., 2010	Cat	Congenital	Day 0	Binaural feature sensitivity is maintained
				Coarse tonotopy maintained
Kral et al., 2001	Cat	Congenital	Day 0	Coarse tonotopy maintained
Hartmann et al., 1997	Cat	Congenital	Day 0	Coarse tonotopy maintained
Fallon et al., 2009	Cat	Ototoxic	Day 17	Tonotopy is lost
Kotak et al., 2007	Gerbil	Cochlear ablation	Day 10	No LTP in layer V neurons

LTP, Long-term potentiation; A1, Primary auditory cortex.

In hearing animals, supergranular layers project to higher-order areas of auditory cortex. The presence of supergranular activity in electrically-stimulated deaf animals suggests that feed-forward connections persist between A1 and secondary auditory areas in deaf animals, at least early in development. Feedback projections from these higher-order auditory areas primarily target the deep layers of A1 (Rouiller et al., 1991). Inactivity in the infragranular layers of deaf A1 suggests that these feedback projections and the associated top-down modulation of activity in A1 do not develop in deaf animals (Raizada and Grossberg, 2003). In support of this idea, *in-vitro* electrophysiological examination of hearing-deprived auditory cortex has demonstrated that layer V neurons are incapable of undergoing the sort of long-term potentiation that normally underlies synaptic plasticity (Kotak et al., 2007).

Functional changes in the primary auditory cortex of congenitally deaf animals have been explored using *in vitro* electrophysiological techniques, as well as through the introduction of peripheral electrical stimulation via a cochlear implant. Multi-unit recordings from deaf A1 show slightly increased spontaneous firing rates when compared to hearing animals, which may reflect upregulated spontaneous activity in thalamic inputs (Kral et al., 2003). Additionally, the excitability of A1 neurons has been shown to increase following deprivation of afferent activity, while inhibition is decreased (Raggio and Schreiner, 1999; Kotak et al., 2005; Kral et al., 2005). Together, these results suggest that cortical neurons favor excitability, likely as a response to reduced cochlear excitation. However, when driven via electrical stimulation, evoked neural activity is decreased in congenitally deaf animals compared to hearing controls (Kral et al., 2005).

Despite changes in the rate of activity, the rudimentary features of A1 neuron responses appear to be present in congenitally deaf animals, despite a complete, and in some cases long-term lack of stimulus-evoked neural activity. For example, the rate-intenisty and latency-intensity functions of electrically-stimulated deaf A1 neurons are similar to those of hearing animals (Raggio and Schreiner, 1994, 1999). Additionally, A1 neurons from congenitally deaf animals demonstrate rudimentary binaural feature sensitivity (Tillein et al., 2010). Interestingly, there are no reports of changes in the temporal processing of electrically stimulated A1 neurons, despite changes in downstream structures (see above).

As in the IC, the auditory cortex of congenitally deaf animals maintains a rudimentary representation of tonotopy, even after extensive periods of hearing loss (Hartmann et al., 1997; Shepherd et al., 1997; Kral et al., 2001, 2002; Tillein et al., 2010), with an activation area similar to hearing controls (Kral et al., 2005). Conversely, neonatally deafened animals show a near-complete loss of tonotopic organization and a rostro-caudal spread of activation in A1 (Raggio and Schreiner, 1999; Fallon et al., 2009). Tonotopic organization of the IC remains following neonatal deafening, and thalamocortical projections to A1 have been shown to be relatively normal in deafened animals (Stanton and Harrison, 2000), suggesting that these differences in A1 tonotopy are the result of reorganization at the level of the thalamus or of A1 itself, serving to increase the overlap between adjacent basilar membrane representations.

## Cross-modal reorganization following deafness

Genetic blueprints play a significant role in the establishment of rudimentary organization throughout the auditory system prior to the onset of hearing. For example, molecular guidanace cues establish tonotopy in the cochlear nucleus in the absence of stimulus-related activity (Kandler et al., 2009), and ectopic projections from the cochlear nucleus to the superior olive are established before the onset of cochlear function (Kitzes et al., 1995; Russell and Moore, 1995). In hearing animals, this organization undergoes stimulus-evoked, activity-dependent refinement, such that adult-like perception is achieved only after hearing onset. As with other sensory systems, congenital deprivation results in an immature system that appears to persist for some time following the normal point of hearing onset. However, if sensory input is not restored before the end of the sensitive period for normal development, many auditory structures may be recruited by another sensory modality. This cross-modal reorganization of cortical structures is thought to underlie behavioral enhancements observed in the remaining sensory modalities of both animal models (e.g., Lomber et al., 2010), and of humans (see Bavelier et al., 2006 for a review).

In hearing animals, the response properties of A1 neurons remain dynamic into adulthood, undergoing rapid changes in order to optimize auditory perception. For example, animals trained to detect a tone of a particular frequency within a complex soundscape show facilitated processing for that frequency in A1 (Fritz et al., 2003). Despite evidence that primary sensory areas are *capable* of processing information from remaining sensory modalities when that information is introduced via surgical manipulation of afferent inputs (Frost and Metin, 1985; Sur et al., 1988; Ptito et al., 2001), crossmodal reorganization in the primary auditory cortex following congenital and early-onset deafness remains a contentious issue. Rebillard and colleagues (1977) reported recording visually-evoked responses to flashes of a stroboscopic light in the primary auditory cortex of both congenitally deaf and early-deafened cats. However, other researchers report an absence of neurons in A1 that are responsive to light flashes or illuminated bars (Stewart and Starr, 1970; Kral et al., 2003). This has led to the belief that A1 is not susceptible to crossmodal reorganization following sensory deprivation. This is in accordance with research in the visual system; congenital blindness leads to the processing of auditory stimuli in areas of cortex which normally process visual information in both cats (Rauschecker and Korte, 1993) and humans (Röder et al., 2000). However, cross-modal reorganization is limited to higher-order visual areas, with no change in primary visual cortex (Yaka et al., 1999; Weeks et al., 2000). Kral and colleagues (2003) also investigated whether cells in deaf A1 were responsive to somatosensory stimulation, finding none that responded to direct stimulation by a cotton pad applied to various parts of the head and body, or to puffs of air directed toward the face of the animal. However, more recent studies in early- (Meredith and Allman, 2012) and late-deaf ferrets (Allman et al., 2009) have found evidence of neurons in core auditory areas, including A1, that are responsive to strokes and taps from brushes and Semmes-Weinstein filaments, as well as puffs of air. In these latter studies, crossmodally activated neurons tended to have large, bilateral receptive fields that were not somatotopically organized. Anatomical tracer injections demonstrated that the pattern of projections between somatosensory areas and A1 in reorganized deaf animals does not differ from the pattern present in hearing animals, suggesting that crossmodal activity does not rely upon the formation of novel projections (Meredith and Allman, 2012). Thus, contradictory data exist with respect to crossmodal reorganization between deaf A1 and both the visual and somatosensory systems. While a number of factors may be involved in these discordant data, a likely candidate involves the anesthetic regimens used. Studies failing to find crossmodal activation of A1 (Stewart and Starr, 1970; Kral et al., 2003) relied on halothane anesthesia, while those demonstrating A1 neurons responsive to non-auditory stimulation used infusions of pentobarbitol (Rebillard et al., 1977), or ketamine and acepromazine (Allman et al., 2009; Meredith and Allman, 2012). Since anesthetics are known to vary in their physiological effects (e.g., Albrecht et al., 1977; Schettini, 1980), it is entirely possible that the presence of crossmodal activation in A1 may be differentially affected by the anesthetic used. Beyond the single animal examined by Rebillard and colleagues (1977), it remains to be seen whether visually-evoked activity can be recorded in deaf A1 under appropriate anesthetic conditions.

Unlike A1, there is convincing evidence that higher-order auditory areas process non-auditory stimuli in deaf animals. For example, it has been demonstrated that recruitment of auditory areas typically involved in sound localization, including the posterior auditory field (PAF; Lomber et al., 2010), and the auditory field of the anterior ectosylvian sulcus (FAES; Meredith et al., 2011), underlies enhanced peripheral localization of visual stimuli in deaf animals. In each of these cases, deaf cats were shown to more accurately detect the location of a small LED light source in the periphery of their visual field than did hearing cats. When PAF was reversibly deactivated (Lomber et al., 2010), deaf cats were no better than hearing cats at this task. Interestingly, when FAES was deactivated in the same manner (Meredith et al., 2011), the accuracy of deaf cats fell to well below that of normal cats, suggesting that deaf FAES is involved in visual target detection *in lieu of*, rather than in addition to the visual cortical area normally involved with this task. The dorsal zone (DZ) of the auditory cortex, which lies adjacent to the visual motion processing regions of the middle suprasylvian sulcus (Lomber, 2001), has been shown to mediate enhanced visual motion sensitivity in deaf animals (Lomber et al., 2010). Deaf cats outperformed hearing controls on a two-alternative forced choice task designed to determine their threshold for visual motion detection. However, the thresholds of the two groups were no different following deactivation of DZ. Finally, neurons in the anterior auditory field (AAF) of deaf animals have been shown to encode somatosensory cues from low-threshold hair receptors stimulated by a soft brush or calibrated filament, as well as movement characteristics of visual stimuli, including their velocity and direction (Meredith and Lomber, 2011).

How the sort of cross-modal reorganization described above might occur remains an issue of some debate (see Bavelier and Neville, 2002, for review). Rauschecker (1995) described several possible cortical mechanisms, including unmasking of silent inputs, stabilization of normally transient connections, sprouting of new axons, or by some combination of these processes. Indeed, anatomical studies have demonstrated that cortical sensory areas are connected both directly (Falchier et al., 2002; Rockland and Ojima, 2003; Hall and Lomber, 2008; Allman et al., 2009; Meredith and Allman, 2012) as well as via multimodal cortical areas (Cappe and Barone, 2005). Thus, it is possible that intermodal connections that are normally latent or transient may underlie reorganization. Such reorganization is often examined using tracer injections designed to determine whether the number of axons connecting sensory areas is increased following deafness. However, intersensory connections might also be strengthened via increases in dendritic branching and synapse number (with or without a change in axonal number). Thus, anterograde tracing and analysis of changes in the number of terminal boutons would provide a fuller insight into the role of intracortical connections in cross-modal plasticity. Conversely, it has also been suggested that cortical reorganization may result from changes in subcortical inputs (Allman et al., 2009). For example, both the cochlear nucleus (Shore and Zhou, 2006) and inferior colliculus (Aitkin et al., 1981) have been shown to respond to somatosensory inputs in hearing animals, and this response is enhanced following hearing loss (Shore et al., 2008; Zeng et al., 2012). In the absence of auditory input, subcortical nuclei may respond to inputs from other sensory modalities, and the reorganization of auditory cortex may simply reflect upstream processing of these changes. Cortical and subcortical mechanisms for reorganization are by no means mutually exclusive; it is likely that cross-modal plasticity involves some combination of mechanisms that depends, at least in part, on the nature of the hearing impairment, the timing of auditory deprivation, and the replacement sensory modality involved.

## Conclusions

The absence of auditory input that accompanies hearing impairment causes long term changes to the structure and function of the auditory system. The exact nature of these changes depends upon factors such as the etiology and onset time of the impairment, and can have significant developmental and psychosocial consequences. Interventions including amplification and cochlear implantation may mediate these consequences, but each depends critically on the integrity and function of remaining auditory structures. Studies undertaken in deaf animal models have provided much of what is known about the function of the deaf auditory system, and have informed the development and design of hearing prostheses. Perhaps most interestingly, these studies have informed our understanding of sensitive periods in development, and their role in functional recovery following the provision of a hearing aid and/or cochlear implant. The animal studies described herein illustrate the importance of early intervention both in terms of minimizing structural and functional damage within auditory structures, as well as recovering auditory cortical areas that might otherwise be recruited by other sensory systems. However, the effects of deafness on higher-order cortical areas and the exact mechanism(s) underlying cross-modal plasticity are not yet fully understood. Thus, research using animal models will continue to inform our understanding of the far-reaching consequences of deafness as the field moves forward.

### Conflict of interest statement

The authors declare that the research was conducted in the absence of any commercial or financial relationships that could be construed as a potential conflict of interest.
